# Ecosystem-based adaptation for increased agricultural productivity by smallholder farmers in Nepal

**DOI:** 10.1371/journal.pone.0269586

**Published:** 2022-06-14

**Authors:** Kiran Bhusal, Erica Udas, Laxmi Dutt Bhatta

**Affiliations:** 1 Center for Environmental and Agricultural Policy Research Extension and Development (CEAPRED), Kathmandu, Nepal; 2 International Centre for Integrated Mountain Development (ICIMOD), Kathmandu, Nepal; 3 Institute of Forestry, Tribhuwan University, Kirtipur, Nepal; PLOS ONE, UNITED KINGDOM

## Abstract

The impacts of climate change are evident in the agriculture sector globally. These impacts are more severe and pronounced in a mountainous country like Nepal due to the high reliance on agro-economy and subsistence-based livelihoods by smallholder farmers that increase vulnerability and risks. Several ecosystem-based adaptation measures have proved to build the adaptive capacity of both agro-ecosystems and smallholder farmers by offering simple and affordable technologies however, these are yet to be prioritized by policy and programs for scaling. In this paper, we provide science-based evidence to traditionally used practices, such as *jholmal* (locally prepared bio-fertilizer and pesticides) and straw mulching by comparing their efficacy in terms of yield and reduction in disease pest infestation. The study was conducted in Kavre district of Nepal during 2017 and 2018 using participatory on-farm field trials for *jholmal* and straw mulching designed separately with Randomized Complete Block Design for selected vegetable crops like bitter gourd and tomato. The application of *jholmal* showed significant increase in bitter gourd yield both at the foothill and hilltop sites compared to the farmer’s business usual practice (in 2017 and 2018, bitter gourd yield increased by 30.5% and 31.1% in foothill, while 26.6% and 28.7% in hilltops respectively). Further, a significant reduction on fruit infestation was observed in *jholmal* treated plots. Similarly, there was increase in tomato yield when straw mulch was used compared to the non-mulched trials (in 2017 and 2018, tomato yield increased by 16.5% and 20.3% respectively). These findings suggest that traditionally used practices have scientific basis and offer simple, affordable and climate friendly practices to improve the health of agro-ecosystem while supporting smallholder farmers to adapt to adverse impacts of climate change and build socio-ecological resilience. These practices can be also customized depending on the local context for wider adoption and scaling across Nepal and elsewhere as ecosystem-based adaptation measures for smallholder farmers.

## Introduction

Mountains, covering one-fifth of the world’s land area, are vulnerable to the impacts of climate change and are disproportionately affected due to heterogeneous ecosystem types that are complex and highly diverse [1–3]. It is projected that temperatures in the mountains across the Hindu Kush Himalaya (HKH) region will increase beyond 2°C on average by 2050, and this is expected to be even more at higher elevations, resulting in the loss of one-third of glaciers by 2100 [3]. Nepal is located in the HKH region and is predominantly mountainous, with about 43% of its total land area categorized as hills [4]. The varied landscapes across the hilly region provide niche micro-climatic habitats favourable to a multitude of ecological zones and diverse farming systems.

Agriculture has been a priority of Nepal’s government since the formulation of the Fifth Five-Year Plan (1975–1980), after which the focus was more on irrigation, introduction of new seeds of high-yielding varieties, and the use of synthetic fertilizers and insecticides to increase production. Before this, the farmers use to practice mostly subsistence oriented traditional farming system using limited locally available natural resources [5]. Such traditional practices–land tillage using livestock for ploughing, use of livestock manure (dung and urine) as bio-fertilizer and bio-pesticide, straw mulching, protection of forests around springs, choice of crops for various agro-ecological systems, local selection of quality seeds and planting season–all came from years of experience and traditional knowledge handed over from one generation to another. Many farmers in Nepal are still subsistence based and do continue to adopt these traditional practices, using local knowledge integrated with scientific evidence to increase crop productivity and adapt to a changing socio-ecological context, including climate change [6, 7].

To reap immediate and direct benefits from the agro-ecological systems in a changing climate, the smallholder farmers in Nepal should focus on sustainable farm management practices that can help them manage climate risks and strengthen adaptive capacity [8]. The traditional knowledge-based farm management practices that are being adopted by the smallholder farmers are ecosystem based and include use of local resources and knowledge, for example- use of *jholmal* (a liquid bio-fertilizer and bio-pesticide), bio-char, farmyard manure, composting, mulching, cover cropping, contour farming, strip cropping, and conservation agriculture practices, along with bioengineering techniques that enhance soil fertility and control erosion [7, 9–11]. However, the use of such traditional practices largely declined over past decades because of agricultural intensification, labour shortage due to increased outmigration, access to road leading to reliance on external resources and decreased availability of local plant resources [10]. On the other hand, to adapt to climate adversities and build resilience of socio-ecological systems, various farm management practices use ecosystem-based adaptation approach as a foundation. For example- climate smart agricultural practices and resilience building solutions in the HKH region have been promoting efficient use of water (water harvesting, storage, source protection and efficient use), soil nutrient management (bio fertilizer and pesticides, farmyard manure, safe farm inputs, reduce nutrient leaching and stabilization of soil erosion), crop diversification (multiple cropping, crop rotation, use of local or climate tolerant varieties) including the use of information and communications technology (agro-advisory services, weather forecast, market price and crop insurance) [6, 12]. Although these practices offer local and nature-based solutions, there is no adequate science-based evidence for wider replication and scaling, which is actually hindering the credibility of these local practices as viable solutions for up-and-out scaling elsewhere.

The current paper thus, intends to provide science-based evidence to support the efficacy of traditionally used practices such as *jholmal* and straw mulching by comparing the yield and rate of disease and pest infestation in selected agriculture crops. The use of *jholmal* and straw mulching, both qualify as ecosystem-based adaptation practices for farm management based on a framework and criteria developed by Vignola et al. [13] that considers: a) conservation, restoration or management of ecosystem processes or services; b) improve the ability of crops to maintain crop yields; and c) appropriate for smallholders, all of which help to increase food security and income, utilize traditional/local knowledge, and are comparatively cost effective methods [6, 7, 14, 15].

## Literature review

In Nepal, hill farming systems cover about 40% of the cultivated lands and these are largely practiced by smallholder farmers [16] who are dependent on subsistence-based agriculture often integrated with livestock and forest ecosystem. The smallholder farmers in the country account for more than 80% with an average landholding size of less than 0.5 hectare [17]. Hill agriculture faces several challenges pertinent to ecological fragility (soil erosion, landslides), mountain specificities (inaccessibility, marginality), water scarcity, pronounced cropping seasons, and limited access to agri-extension services, tools and technologies, agricultural inputs, and market opportunities [6, 18–20]. Currently, increase in migration, labour shortage, lack of technological adoption and abandonment of farmland -are leading to decrease in agricultural productivity [21] posing a greater threat to food security and achieving the zero hunger target of the Sustainable Development Goals 2030. One of the reasons for low agricultural productivity is decline in soil fertility, largely due to soil erosion, intensive farming resulting to reduced organic matter and nutrient mining, as well as the improper use of chemical fertilizers and pesticides [22]. It is estimated that 1.7 mm of topsoil is lost each year due to soil erosion, mostly triggered by conventional tillage practices coupled with heavy rainfall on slopes, leading to deterioration in soil properties, and affecting crop yield over time [9, 23]. On the other hand, excessive use of chemical fertilizers adversely affects productivity, water quality, as well as food and nutrition security [9]. The trend of chemical fertilizer and pesticide use in Nepal from 2012/2013 to 2016/2017 shows continuous increase, particularly in vegetable farming, with a sub-standard level of pesticide residue both nationally and internationally [24–27] raising environmental and food safety concerns linked to human health and vitality of agro-ecosystem. Likewise, outmigration of men and youth is another social challenge in the hill agriculture context; particularly in Nepal, 57% of the households are engaged in off-farm employment, providing social and economic remittances, but this has however, resulted in farm labour shortages with increased workload and drudgery for women [28].

Climate change has cascading impacts to the challenges of hill agriculture where many smallholder farmers primarily rely on rain-fed agriculture. So, both short- and long-term climate variability and climate change may have severe impacts on plant growth, plant phenology and yields of crops, vegetables, and fruits, particularly due to shifts in rainfall patterns, prolonged drought, warming induced risks of pest and disease infestation, and altered nutrient cycling [29–32]. Climate uncertainties such as erratic rainfall events, flash floods and hailstorms also seriously exacerbate the threats to agro-ecological ecosystems and the services they deliver, ultimately impeding the lives and livelihoods of smallholder farmers and the mountain communities. Therefore, strengthening adaptive capacities of smallholder farmers to absorb, adapt and transform to climate shocks and extremes is crucial while also acknowledging the existing traditional and local knowledge system for complementing it with scientific evidence. In this context, an ecosystem-based approach to climate change adaptation offers cost-effective solutions to sustainably manage and restore smallholder hill agriculture systems as a part of the overall adaptation strategy.

Ecosystem based adaptation is defined as “sustainable management, conservation and restoration of ecosystems, as part of an overall adaptation strategy that takes into account the multiple social, economic, and cultural co-benefits for local communities” [33]. It is being widely used as sustainable, cost-effective solution to generate social, economic, and environmental co-benefits and adapt to climate change impacts [34–37]. Several government and multilateral organizations, I/NGOs are promoting ecosystem-based adaptation measures to help smallholder farmers adapt to climate change and build resilience [38–40]. The farmers in Nepal have been using indigenous and traditional knowledge for generations to conserve forests, agriculture lands, water sources, wildlife, and fisheries [10, 41]. Such traditionally rooted ecosystem-based management practices in agricultural systems can help smallholder farmers adapt to adverse impacts of climate change by providing both on-site (farm level) and off-site (landscape level) benefits [13].

## Materials and methods

### Study area

**Fig 1:**
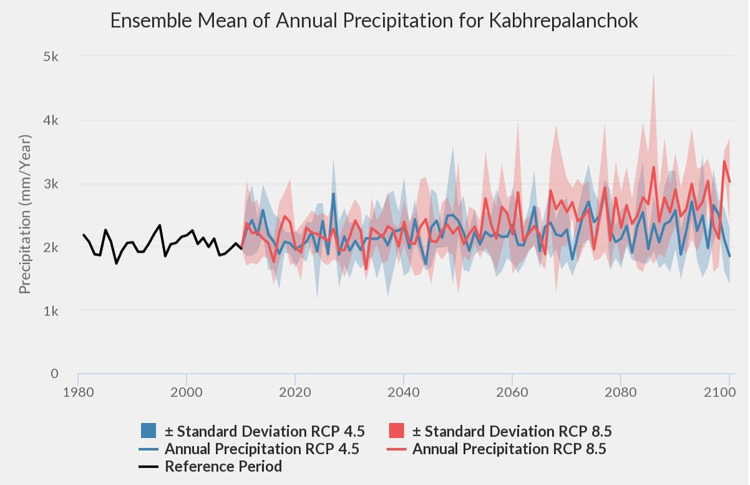
Ensemble mean of annual precipitation from Kavre district Nepal. (Source: MoFE, 2019).

**Fig 2:**
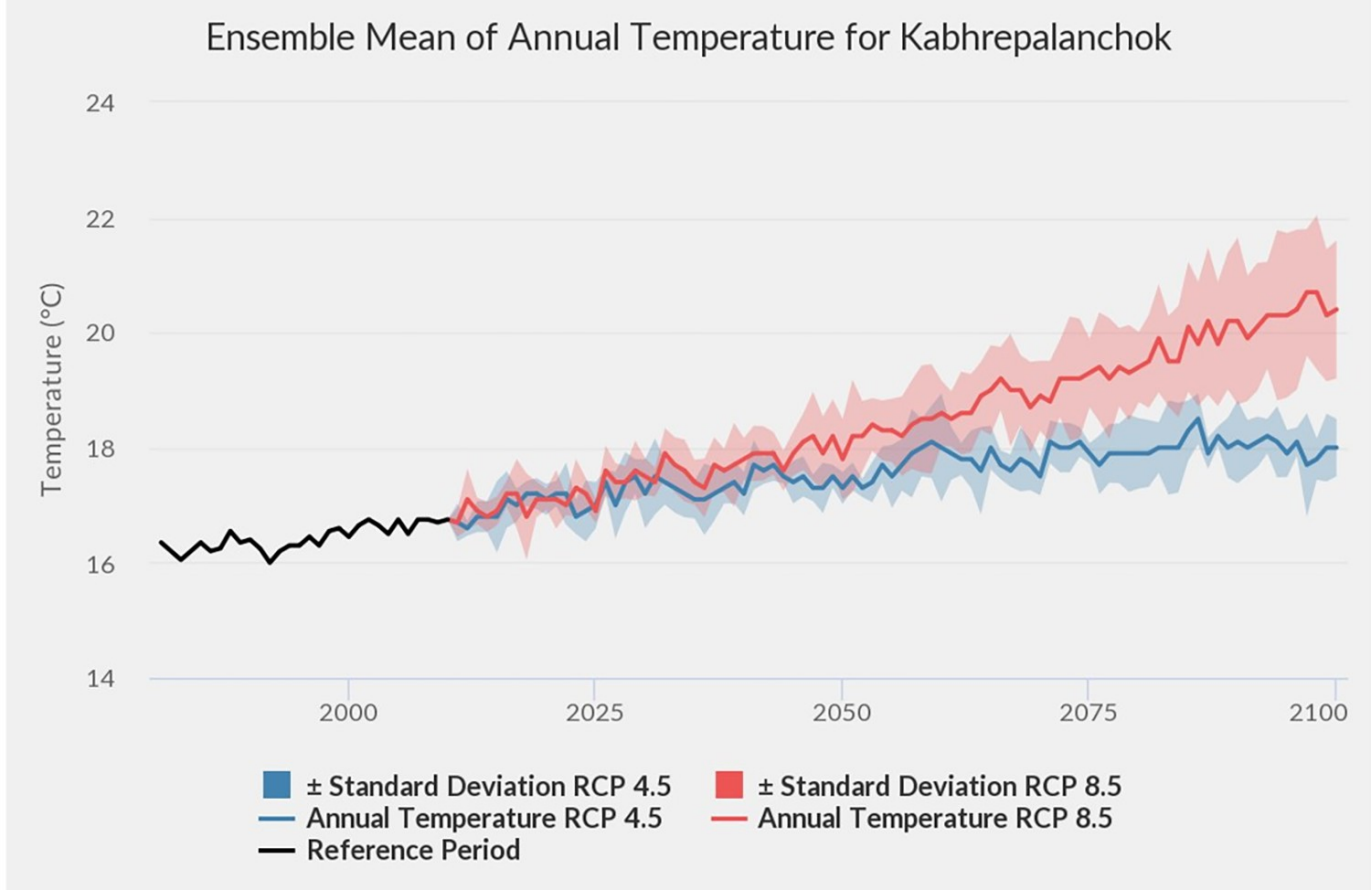
Ensemble mean of annual temperature from Kavre district Nepal. (Source: MoFE, 2019).

Kavre district is well known for its vegetable production. Much of this produce is supplied to the capital, Kathmandu which is located about 30 km to the west. About 65.3% of the area in the district is under the subtropical climatic zone, representing the dominant climatic zone of the mid hills of Nepal, while the rest belongs to tropical and temperate climatic zones [42]. The average annual temperature in the district is 9.1°C in January and 21.7°C in June depending upon the altitude, while annual rainfall ranges between 1500–2000 mm [7]. The historical climate data of Kavre district from 1971 to 2014 shows increasing temperature and decreasing precipitation in all four major seasons as indicated in Table 1 [43]. During this 45-year period, maximum temperature increased by 1.9°C and minimum temperature increased by 0.9°C, whereas precipitation decreased by 8% (110 mm), although the latter is not statistically significant [43]. The future climate projections in Kavre by MoFE [44] suggest hotter and wetter climate (Figs 1 and 2) which on the one hand may favour plant production, while increase the risks of insect pest infestation on the other hand.

**Table 1:** Change in temperature and precipitation from 1971 to 2014 in Kavre district.

Seasons	Precipitation (mm/year)	Max. temperature (°C/year)	Min. temperature (°C/year)
Winter	-0.09	0.037**	0.015**
Pre-monsoon	-0.03	0.039**	0.016**
Monsoon	-2.44	0.043***	0.023***
Post monsoon	-0.18	0.043***	0.017**
Annual	-2.45	0.042**	0.020**

Source: DHM 2017

** is significant at 95% and

*** is significant at 99.9%

For the current study, Kavre district was purposively selected as it represents an intensive vegetable cultivation pocket area [45] and nearly 72% of agricultural land holdings are under vegetable cultivation [46]. There is massive application of pesticides to control various insect pests and diseases mainly in potato, tomato, rice, cauliflower, eggplant, chilli, beans, gourds, wheat and mustard [47]. High pesticide use has contaminated soils, water (ground and surface) and also increased exposure and health risks of farmers as well as consumers [27, 48]. Farmers in Kavre reported acute health related symptoms such as headache, skin and eye irritation, chest pain, and throat discomfort leading to increased out of pocket expenditure on health care [48].

**Fig 3:**
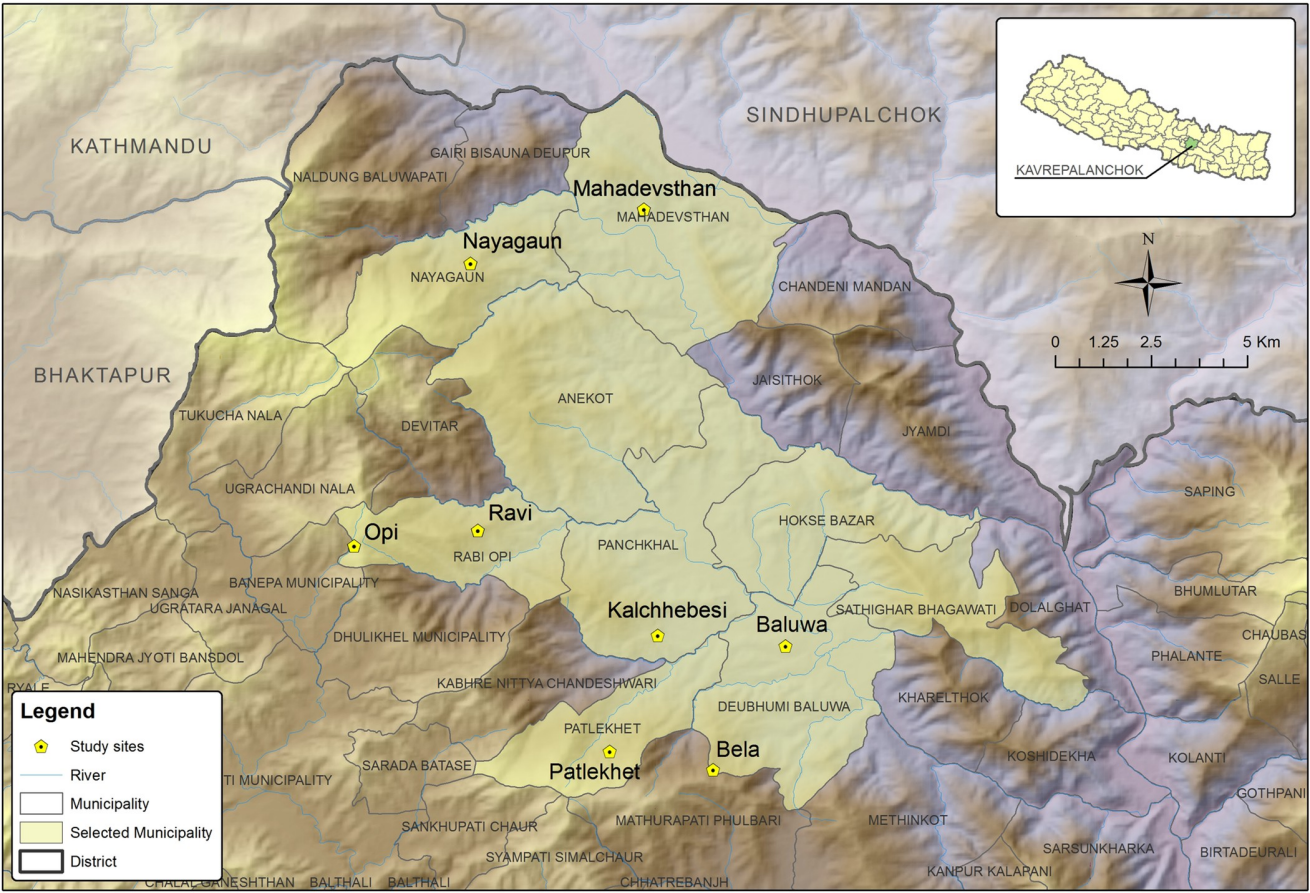
Study sites at Kavre district of Nepal. (Source: ICIMOD).

The study area in Kavre covers a total of eight sites along an altitudinal gradient to represent site heterogeneity, with four sites representing the foothills/low land at altitudes ranging from 875–980 masl and the other four sites representing hilltops at altitude ranging from 1235–1415 masl (Fig 3 and Table 2). Many of these sites are drought prone areas of Kavre district as declared by the Government of Nepal [49]. Therefore, to address the issues of high pesticide use and prevailing drought in the district, *jholmal* and straw mulching were introduced as ecosystem-based adaptation measures that could serve as alternatives to widespread chemical pesticide use and to improve water retention, thereby minimizing health and environmental risks, including the impacts of climate change.

**Table 2:** Description of the eight study sites at Kavre.

#	Name of selected sites	Soil type	Agro ecological zone	Altitude (masl)
1	Opi (OP)	Loam and sandy loam	Hilltops	1415
2	Pathlekhet (PA)	Loam	Hilltops	1320
3	Nayagaun (NA)	Loam and sandy loam	Hilltops	1235
4	Bela (BE)	Loam	Hilltops	1297
5	Rabi (RA)	Sandy loam and loam	Foothills	877
6	Kalchebeshi (KA)	Loam and sandy clay loam	Foothills	980
7	Mahadevsthan (MA)	Loam and sandy loam	Foothills	892
8	Baluwa (BA)	Loam and sandy loam	Foothills	875

### Experimental design

On-farm research trials on application of *jholmal*-1, 2 and 3 was carried out in eight selected study sites at the hilltops and foothills of Kavre district, whereas trials on straw mulching were carried out in four selected sites at the hilltops. Altogether, 32 lead farmers from the respective farmers’ groups were chosen through a participatory meeting to conduct the research trials on *jholmal* and straw mulching (16 each). A Randomized Complete Block Design (RCBD) was used for the experimental design where the experimental and control trials for both *jholmal* and straw mulching were established adjacent to each other to maintain homogeneity in site quality, slope, and aspect. However, potential inter specific site heterogeneity within the trials was neglected. The details on experimental design and treatments for *jholmal* and straw mulching are elaborated below.

### Jholmal

**Fig 4:**
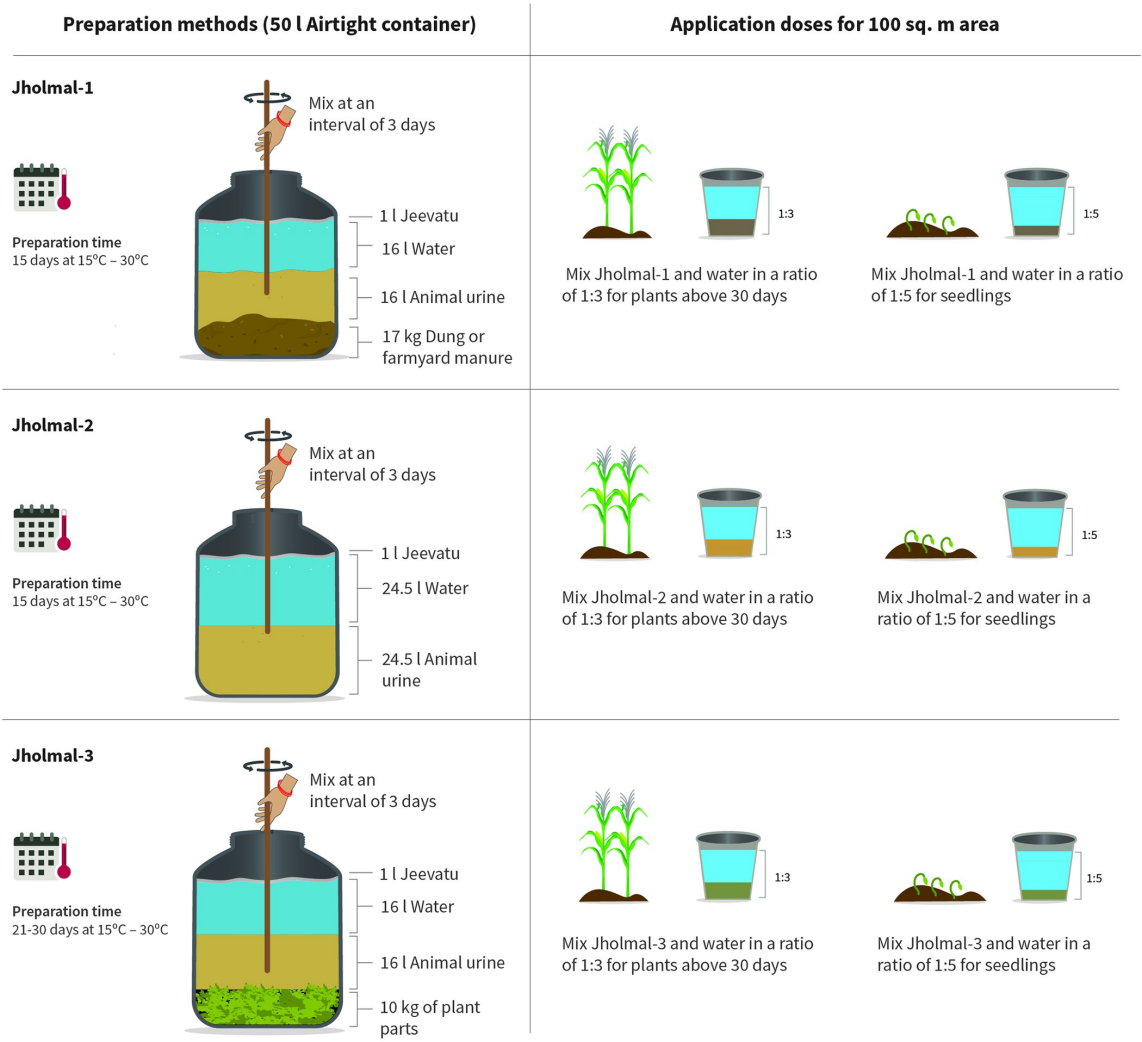
Preparation and application of different *jholmal* formulations.

*Jholmal* is a traditionally used, homemade liquid bio fertilizer and pesticide prepared by mixing animal urine, Farmyard manure (FYM), locally available botanical plants with insect repelling properties, and water at definite proportions [50]. Cow urine and selected botanicals have traditionally been used to cure diseases and pest in agriculture systems [51]. Such traditional knowledge-based practice was customized to develop three different types of *jholmal* formulations, namely *jholmal*-1, 2 and 3 by CEAPRED and ICIMOD. *Jholmal*-1 is used as bio fertilizer while *jholmal*-2 and *jholmal*-3 are used as bio pesticides. To generate science-based evidence, on-farm experimental trials were conducted at these eight sites using these formulations. Fig 4 illustrates the method of *jholmal* preparation and their application doses in general.

The effect of *jholmal*-1, 2 and 3 on yield of bitter gourd (*Momordica charantia* L.) was studied in 2017–2018 during its normal growing season (March-September). The seeds were directly sown on the experimental and control trial plots of 100 m^2^ each during early March. Bitter gourd was selected for the study because it is commercially grown and widely consumed, and it is highly affected by fruit fly infestation compared to other horticultural crops like fruits and solanaceous vegetables [52]. Altogether, 16 lead farmers from eight sites were selected based on their willingness to participate in the research on *jholmal* application. Among the selected lead farmers 8 were from the foothills and 8 were from hilltops. Each farmer was considered as one replication with experimental and control trial plots.

The *jholmal* formulations were applied systematically in the experimental trial plots at the rate of 12 litres *jholmal*-1 plus 12 litres *jholmal*-2 plus 125 kg FYM (*jholmal*-2 treatment), or 12 litre *jholmal*-1 plus 12 litre *jholmal*-3 plus 125 kg FYM (*jholmal*-3 treatment) per 100 m^2^ area. The *jholmal*-2 treatment and *jholmal*-3 treatment differed in terms of application of *jholmal*-2 and *jholmal*-3 while *jholmal*-1 and FYM were common to both treatments. Application of these *jholmal* formulations in the experimental trial plots was carried out at 15-day intervals after the germination of the seedlings to ensure nutrient availability and insect/pest repellence. The farmers’ business as usual practice included use of 250 kg FYM, which served as control trial plots. These three different treatments are listed below:

*Jholmal*-2 treated: 12 L *jholmal*-1 + 12 L *jholmal*-2 + 125 kg FYM
*Jholmal*-3 treated: 12 L *jholmal*-1 + 12 L *jholmal*-3 + 125 kg FYM
Farmers’ business as usual practice: 250 kg FYM

–Nearly 32 harvests of bitter gourd were carried out at an interval of 4 days with a standard desirable size after the fruit colour turned from pale green to dark green. A digital weighing machine was used to weigh the harvest with and without fruit infestation from experimental and control trial plots separately. The cumulative harvested weight throughout the season was recorded in a file by the lead farmers under close supervision of agriculture technicians, which was used to determine total yield and total fruit infested.

### Mulching

The practice of mulch application for vegetable production goes back thousands of years [53, 54]. Mulching involves covering a layer of material on the soil around the crop to maintain proper growing environment and improve crop productivity. The primary purposes for using mulches are to suppress weeds, conserve water, reduce soil erosion and control soil temperature fluctuation [38]. Straw mulching was used in experimental trial plots of tomato (*Solanum lycopersicum* L.), while the control plots were left uncovered. Tomato was chosen for this research trial as it is a major year-round vegetable crop with high marketing possibilities in the mid hills of Nepal [55]. Tomatoes grown during the early spring in the hills are sold as off-season vegetables and fetch a higher price in the markets [55], providing higher returns to smallholder farmers.

Altogether, 16 farmers from four trial sites were selected from areas prone to erosion, surface runoff, and water scarcity, and farmers’ willingness to conduct the research on their land. Each farmer was considered as one replication with experimental and control trial plots. These sites had very limited access to water for irrigation during the early spring season; hence this experiment was carried out to generate science-based evidence for coping with the existing problem of water scarcity.

Harvests of the tomatoes were carried out at a standard desirable size at an interval of 4 days when the fruit colour turned from green to pinkish red (turning stage) for a total of 46 times in one growing season from each trial plots. A digital weighing machine was used to weigh the harvested tomatoes and records of each harvest from experimental and control trial plots were kept separately by the lead farmers under close supervision of the agriculture technicians to determine total yield.

### Data analysis

The data on yield and fruit infestation were recorded from experimental and control trial plots and later extrapolated to tonnes per hectare. The primary data obtained from research trials were first tabulated in MS excel. The data were then cleaned and processed for analyses in R 3.0.3 software using the R package ‘agricolae’ version 1.1–8. The effect of treatment types on yield were analysed using analysis of variance (ANOVA). The comparisons of means between the treatments were differentiated using Duncan’s Multiple Range Test (DMRT) [56]. All the figures and graphs were prepared using MS Excel.

## Results and discussion

### Effect of *jholmal* on bitter gourd yield

Highly significant differences in yield were observed due to the application of *jholmal* in bitter gourd compared to the farmers’ business as usual practice (Table 3). The yield of the bitter gourd in trials with *jholmal*-3 increased in both years by 30.5% and 31.1% in the foothills and by 26.6% and 28.7% in hilltops compared to the farmers’ business as usual practice. Similarly, *jholmal*-2 treated plots also showed increased yield in both years by 12.2% and 9.79% in the foothills and 10.9% and 13.8% at the hilltops compared to the farmers’ business as usual practice. Our results showed slightly higher yield of bitter gourd as compared to what Subedi et al. [7] reported as 15.4 ton/ha when they applied 4.8 litres *jholmal*-1 and *jholmal*-3 each, 7.2 litres *jholmal*-2 and 120 kg FYM in 100 m^2^ research trials in 2016. On the other hand, Arora et al. [57] have reported increase in tomato yield by application of fermented botanicals in cow urine. The increase in yield due to the application of different *jholmal* formulations may be because of faster supply of nutrients with foliar application that supplement plant growth by overcoming temporary nutrient deficiencies [58] and it also work as a plant growth regulator [57]. Further, *jholmal* use may help to increase microbial activities on soil which in turn releases nutrients for crop and hence enhances yield. Gajjela et al. [58] have reported that increase in soil microbe numbers helps to loosen the soil pores and increases nutrient release that can be easily up taken by crops. Yadav et al. [59] also reported that liquid fertilizers are important for agriculture because their beneficial microbes can enhance plant growth and nutrient uptake through solubilisation of phosphorus, potassium, and zinc as well as help in nitrogen fixation. In Table 4, the pH and nutrient content of the different *jholmal* formulations are illustrated after lab testing.

**Table 3:** Effect of different treatments on bitter gourd yield at Kavre, Nepal.

Treatments	Foothills		Hilltops	
	Yield (t/ha)			
	2017	2018	2017	2018
*Jholmal*-2	20.2 ± 0.8 ^b^	21.30 ± 1.1 ^b^	18.3 ± 1.6 ^b^	20.6 ± 2.3 ^b^
*Jholmal*-3	23.5 ± 1.6 ^a^	25.45 ± 1.4 ^a^	20.9 ± 1.7 ^a^	23.3 ± 1.2 ^a^
Farmers’ business as usual practice	18.0 ± 1.3 ^c^	19.40 ± 1.8 ^c^	16.5 ± 1.4 ^c^	18.1 ± 1.9 ^c^
Significance	***	***	***	***

Note: The alphabetic superscripts are significant at p ≤0.001 by Duncan’s Multiple Range Tests

**Table 4:** pH and nutrient content in different types of *jholmal*.

*Jholmal* type	pH	N %	P_2_O_5_%	K_2_O %	Organic carbon %
*Jholmal*-1	7.85	0.85	0.10	0.85	0.76
*Jholmal*-2	7.66	0.75	0.09	0.75	0.26
*Jholmal*-3	7.55	0.70	0.08	0.70	0.22

**Fig 5:**
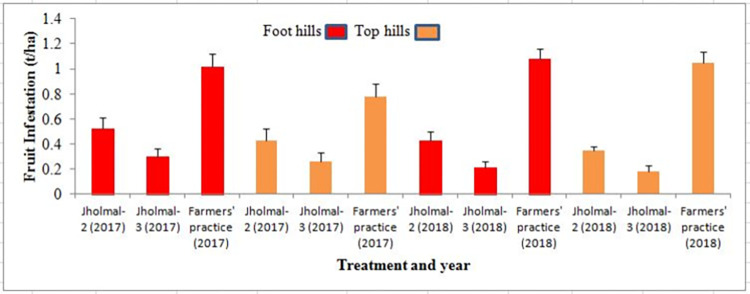
Fruit fly infestation under different treatments in two agro ecological zones.

On the other hand, highest yield was recorded from the trials with *jholmal*-3 treated plots compared to that of *jholmal*-2, although the major nutrient content in different types of *jholmal* formulations was found to be similar (Table 4). However, the main reason behind higher yield in *jholmal*-3 treated plots could be a result of less fruit infestation (Fig 5) which can be attributed to its antifeedant and pest repellent properties, particularly from the selected botanicals used in *jholmal*-3 preparation. Matharu et al. [60] also reported that homemade liquid pesticides with botanicals possess pest repellence and antifeedant properties and can thus be an alternative to chemical pesticides.

The yield of bitter gourd was higher in the foothills compared to the hilltops, which may be due the warmer weather condition in the foothills. Generally, temperature falls/lapses as altitude increases and an average environmental lapse rate for the earth is 0.6 to 0.7^0^ C for every 100 m increase in elevation [61]. This means that foothills are much warmer than hilltops and favour plant growth and yield. The optimum temperature requirement for bitter gourd growth and development is 25−30^0^ C [62] and being a warm season crop, better growth, earlier fruiting, and higher yields are attained at higher daily mean temperatures as observed by other researchers [63–65]. On the other hand, there is less likelihood of soil nutrient loss through surface runoff and erosion in the foothills compared to the hilltops. Atreya et al. [66] reported higher nutrient loss in hills due to erosion and surface runoff leading to reduction in crop production.

However, in a changing climate context with increase in temperature the insect pest infestation rate would be also high leading to increased crop damage as well as affecting plant growth and development [67–70]. The comparison of pest infestation in the foothills and hilltops in the current study however did not show any statistical significance. This technology is being promoted by several governmental and non-governmental organizations in different mid-hills districts of Nepal. Nevertheless, given the efficacy of *jholmal* in increasing the yield as well as reducing the pest infestation, it could be one of the most suitable ecosystem-based adaptation measures for smallholder farmers and can be scaled up and customized based on the local context.

### Effect of straw mulching on tomato yield

A significant difference in tomato yield was observed in trial plots with straw mulching compared to non-mulched plots (Table 5). The yield of tomato increased by 16.5% and 20.3% in 2017 and 2018, respectively when compared to the non-mulched plots. This result is in line with similar studies which showed increase in yield using organic mulch [71, 72]. We argue that the increased yield in our study sites may be due to reduced soil runoff, weed suppression, provision of nitrogen due to decomposition of mulch, and moisture conservation using mulching. Yu et al. [72] also reported that mulching helps to improve crop growth as well as yield and optimizes water use. As historic climate trends of Kavre and future climate projections show increase in temperature, mulching can be a cost-effective ecosystem-based adaptation measure for smallholder farmers. Additionally, rice straw mulching helps in reduction of soil runoff by up to 18% [10]. Borst and Woodburn [73] also reported 43% reduction in soil runoff using straw as mulch. Organic mulch also helps to suppress weeds [74] and mulches containing low nitrogen (e.g. straw mulch) increase fertility and nutrient level in soil by undergoing decomposition [75].

**Table 5:** Effect of different treatments on tomato yield at Kavre, Nepal.

Treatments	Yield (t/ha)	
	2017	2018
Mulched	18.21 ± 1.5 ^a^	19.17 ±1.9 ^a^
Non mulched	15.63 ± 1.6 ^b^	15.93 ± 2.1 ^b^
Significance	***	***

Note: The alphabetic superscripts are significant at p ≤0.001 by Duncan’s multiple range tests

The increase in yield is one of the reasons behind mulching being popular among smallholder farmers in Kavre and several organizations are promoting the technologies in different mid-hill districts of Nepal. Use of such low-cost mulching techniques to increase tomato yield has not only improved the economy of the farmers but has also changed their mindset towards the use of locally available resources to enhance soil fertility, conserve soil moisture, suppress weeds and reduce soil runoff, which in turn contributes to overall sustainable management of the agro-ecosystem. It has multiple benefits with tremendous potential to be scaled up in Nepal and elsewhere.

### Policy implications and recommendations

Nepal’s National Adaptation Plan (NAP) identified eight sectors negatively impacted from climate change. Agriculture and food security is among those eight sectors, and prioritized as the most vulnerable to climate change. NAP also suggested actions to minimize impacts from climate change through adoption of climate friendly technologies, and land use management practices. Our results indicate that climate friendly small scale technologies in land use management not only reduced the use of chemicals, but also increased soil fertility and production, which is in line with the national policy priority. However, there is a need of incentivizing these smallholder farmers practicing climate friendly innovations in land use and practices.

## Conclusion

*Jholmal* and straw mulching are traditionally used practices by smallholder farmers that offer simple and affordable nature-based solutions for ecosystem-based adaptation. These solutions are largely recommended for smallholder farmers. The findings from the current study showed increased yield of bitter gourd and tomato compared with the farmers’ business as usual practice when *jholmal* and straw mulch were used respectively. The application of *jholmal*-3 prepared with selected locally available botanicals and animal urine and dung was effective in reducing pest infestation and could be an alternative to chemical pesticides. The application of these practices not only increases yield and controls crop pests but also helps smallholder farmers to save input costs (purchase of chemical fertilizers and pesticides), while also ensuring food security by increasing the based income through seasonal and off-seasonal vegetable production. Use of *jholmal* further ensures safe food production and safeguards agro-ecosystem and human health. Similarly, mulching practices conserve soil moisture and minimize soil nutrient runoff for better plant growth and yield. In this regards, *jholmal* and straw mulching proved to be effective EbA measures that increase productivity while reducing the pest infestation and use of pesticides. These measures further amplify local adaptation capacity at scale through the use of locally available resources and technologies that are affordable to smallholder farmers. Therefore, traditional practices combined with scientific knowledge, provide evidence for policy makers and adaptation programmes to promote and scale these solutions across Nepal and elsewhere with customization based on local context.

The findings from this paper open up the avenues to explore transformation of mountain agriculture towards new modes of safe food production by highlighting positive environmental and human health outcomes particularly with the use of *jholmal* as bio-fertilizer and bio-pesticide. The science-based evidence generated for the traditionally used practices revealed increased yield and less pest infestation hence, bringing a strong proof to be promoted as simple and affordable ecosystem-based adaptation solution at scale. Further, there are also potentials for business plan development and market linkages, and government policies should actually encourage investments in such local technologies to have significant positive influence on hill farming system and contribute to the livelihoods of smallholder farmers in Nepal and elsewhere. Additionally, there is also scope for future research related to using appropriate concentration and application interval of *jholmal*, the effect of *jholmal* and mulching on soil fertility and water holding capacity, as well as linkages to human health and soil microbial activities

## Supporting information

S1 File (DOCX)

S2 File (DOCX)
