# Supplementary material for: Ecosystem-based adaptation for increased agricultural productivity by smallholder farmers in Nepal
Source: PLoS One. 2022 Jun 14;17(6):e0269586. doi: 10.1371/journal.pone.0269586 (PMC9197052; doi:10.1371/journal.pone.0269586)
Supplement: S1 File — (DOCX) [file pone.0269586.s001.docx]

Data on Jholmal

| Area | Replications | Treatments | Yield 2017 (t/ha) | Yield 2018 (t/ha) | Fruit infestation 2017 (t/ha) | Fruit infestation 2018 (t/ha) |
| --- | --- | --- | --- | --- | --- | --- |
| Hilltops | r1 | Jholmal-2 | 18.1 | 20.4 | 0.3 | 0.4 |
| Hilltops | r1 | Jholmal-3 | 18.8 | 21.2 | 0.2 | 0.1 |
| Hilltops | r1 | Farmers practice | 14.7 | 16.3 | 1.3 | 0.9 |
| Hilltops | r2 | Jholmal-2 | 17.2 | 19.5 | 0.5 | 0.3 |
| Hilltops | r2 | Jholmal-3 | 21.7 | 24.1 | 0.19 | 0.4 |
| Hilltops | r2 | Farmers practice | 17 | 18.6 | 0.8 | 1.1 |
| Hilltops | r3 | Jholmal-2 | 19.3 | 21.6 | 0.6 | 0.4 |
| Hilltops | r3 | Jholmal-3 | 20 | 22.4 | 0.3 | 0.3 |
| Hilltops | r3 | Farmers practice | 15.9 | 17.5 | 0.9 | 0.8 |
| Hilltops | r4 | Jholmal-2 | 18.4 | 20.7 | 0.4 | 0.5 |
| Hilltops | r4 | Jholmal-3 | 22.9 | 25.3 | 0.3 | 0.4 |
| Hilltops | r4 | Farmers practice | 18.2 | 19.8 | 1.1 | 1.03 |
| Hilltops | r5 | Jholmal-2 | 18.2 | 20.5 | 0.7 | 0.5 |
| Hilltops | r5 | Jholmal-3 | 18.9 | 21.3 | 0.6 | 0.18 |
| Hilltops | r5 | Farmers practice | 14.8 | 16.4 | 1.12 | 0.9 |
| Hilltops | r6 | Jholmal-2 | 17.3 | 19.6 | 0.8 | 0.6 |
| Hilltops | r6 | Jholmal-3 | 21.8 | 24.2 | 0.4 | 0.5 |
| Hilltops | r6 | Farmers practice | 17.1 | 18.7 | 0.9 | 0.82 |
| Hilltops | r7 | Jholmal-2 | 19.4 | 21.7 | 0.48 | 0.5 |
| Hilltops | r7 | Jholmal-3 | 20.1 | 22.5 | 0.3 | 0.16 |
| Hilltops | r7 | Farmers practice | 16 | 17.6 | 1.04 | 0.8 |
| Hilltops | r8 | Jholmal-2 | 18.5 | 20.8 | 0.43 | 0.3 |
| Hilltops | r8 | Jholmal-3 | 23 | 25.4 | 0.14 | 0.3 |
| Hilltops | r8 | Farmers practice | 18.3 | 19.9 | 0.93 | 0.7 |
| Foothills | r9 | Jholmal-2 | 19.75 | 20.85 | 0.5 | 0.6 |
| Foothills | r9 | Jholmal-3 | 23.17 | 25.12 | 0.2 | 0.5 |
| Foothills | r9 | Farmers practice | 17.37 | 18.77 | 0.9 | 1.1 |
| Foothills | r10 | Jholmal-2 | 20.59 | 21.69 | 0.6 | 0.7 |
| Foothills | r10 | Jholmal-3 | 24.05 | 26 | 0.4 | 0.45 |
| Foothills | r10 | Farmers practice | 18.21 | 19.61 | 0.7 | 0.9 |
| Foothills | r11 | Jholmal-2 | 17.74 | 18.84 | 0.6 | 0.75 |
| Foothills | r11 | Jholmal-3 | 20.89 | 22.84 | 0.5 | 0.6 |
| Foothills | r11 | Farmers practice | 15.72 | 17.12 | 0.9 | 1.3 |
| Foothills | r12 | Jholmal-2 | 18.55 | 19.65 | 0.7 | 0.5 |
| Foothills | r12 | Jholmal-3 | 21.7 | 23.65 | 0.36 | 0.4 |
| Foothills | r12 | Farmers practice | 16.53 | 17.93 | 1.3 | 0.89 |
| Foothills | r13 | Jholmal-2 | 21.79 | 22.89 | 0.8 | 0.7 |
| Foothills | r13 | Jholmal-3 | 25.25 | 27.2 | 0.5 | 0.6 |
| Foothills | r13 | Farmers practice | 19.41 | 20.81 | 1.2 | 1.21 |
| Foothills | r14 | Jholmal-2 | 22.69 | 23.79 | 0.5 | 0.6 |
| Foothills | r14 | Jholmal-3 | 26.15 | 28.1 | 0.48 | 0.5 |
| Foothills | r14 | Farmers practice | 20.31 | 21.71 | 1.4 | 1.01 |
| Foothills | r15 | Jholmal-2 | 19.84 | 20.94 | 0.7 | 0.7 |
| Foothills | r15 | Jholmal-3 | 22.99 | 24.94 | 0.5 | 0.6 |
| Foothills | r15 | Farmers practice | 17.82 | 19.22 | 1.2 | 1 |
| Foothills | r16 | Jholmal-2 | 20.65 | 21.75 | 0.6 | 0.6 |
| Foothills | r16 | Jholmal-3 | 23.8 | 25.75 | 0.5 | 0.4 |
| Foothills | r16 | Farmers practice | 18.63 | 20.03 | 1.1 | 0.8 |
