# Supplementary material for: Ecosystem-based adaptation for increased agricultural productivity by smallholder farmers in Nepal
Source: PLoS One. 2022 Jun 14;17(6):e0269586. doi: 10.1371/journal.pone.0269586 (PMC9197052; doi:10.1371/journal.pone.0269586)
Supplement: S2 File — (DOCX) [file pone.0269586.s002.docx]

Data on mulching

| Area | Replications | Treatments | Yield 2017 (t/ha) | Yield 2018 (t/ha) |
| --- | --- | --- | --- | --- |
| Hilltops | r1 | Mulched | 19.23 | 20.11 |
| Hilltops | r1 | Non mulched | 16.21 | 17.81 |
| Hilltops | r2 | Mulched | 15.63 | 18.91 |
| Hilltops | r2 | Non mulched | 14.22 | 15.52 |
| Hilltops | r3 | Mulched | 18.45 | 20.62 |
| Hilltops | r3 | Non mulched | 16.33 | 17.36 |
| Hilltops | r4 | Mulched | 19.34 | 20.67 |
| Hilltops | r4 | Non mulched | 14.57 | 16.63 |
| Hilltops | r5 | Mulched | 18 | 20.81 |
| Hilltops | r5 | Non mulched | 16.34 | 17.84 |
| Hilltops | r6 | Mulched | 17.54 | 20.04 |
| Hilltops | r6 | Non mulched | 15.99 | 17.29 |
| Hilltops | r7 | Mulched | 18.67 | 20.87 |
| Hilltops | r7 | Non mulched | 17.33 | 18.43 |
| Hilltops | r8 | Mulched | 20.89 | 22.19 |
| Hilltops | r8 | Non mulched | 18.1 | 17.4 |
| Hilltops | r9 | Mulched | 18.73 | 18.7 |
| Hilltops | r9 | Non mulched | 11.71 | 14.4 |
| Hilltops | r10 | Mulched | 15.13 | 18.2 |
| Hilltops | r10 | Non mulched | 13.72 | 14.6 |
| Hilltops | r11 | Mulched | 17.95 | 17.6 |
| Hilltops | r11 | Non mulched | 15.83 | 13.9 |
| Hilltops | r12 | Mulched | 18.84 | 17.8 |
| Hilltops | r12 | Non mulched | 14.07 | 14.9 |
| Hilltops | r13 | Mulched | 17.5 | 19.1 |
| Hilltops | r13 | Non mulched | 15.84 | 14.7 |
| Hilltops | r14 | Mulched | 17.04 | 16.3 |
| Hilltops | r14 | Non mulched | 15.49 | 13.6 |
| Hilltops | r15 | Mulched | 18.17 | 16.3 |
| Hilltops | r15 | Non mulched | 16.83 | 15.1 |
| Hilltops | r16 | Mulched | 20.39 | 18.5 |
| Hilltops | r16 | Non mulched | 17.6 | 15.4 |
